# Intestinal Mucosal Mast Cells: Key Modulators of Barrier Function and Homeostasis

**DOI:** 10.3390/cells8020135

**Published:** 2019-02-08

**Authors:** Mercé Albert-Bayo, Irene Paracuellos, Ana M. González-Castro, Amanda Rodríguez-Urrutia, María J. Rodríguez-Lagunas, Carmen Alonso-Cotoner, Javier Santos, María Vicario

**Affiliations:** 1 Laboratory of Translational Mucosal Immunology, Digestive Diseases Research Unit, Vall d’Hebron Institut de Recerca, Department of Gastroenterology, Hospital Universitario Vall d’Hebron, 08035 Barcelona, Spain; merce12795@gmail.com (M.A.-B.); irene.paracuellos@e-campus.uab.cat (I.P.); 2 Department of Psychiatry, Hospital Universitari Vall d’Hebron & Facultat de Medicina, Universitat Autònoma de Barcelona, 08035 Barcelona, Spain; amarodriguez@vhebron.net; 3 Centro de Investigación Biomédica en Red de Salud Mental (CIBERSAM), 28029 Madrid, Spain; 4 Department of Biochemistry and Physiology, Faculty of Pharmacy and Food Sciences, University of Barcelona, 08028 Barcelona, Spain; mjrodriguez@ub.edu; 5 Laboratory of Neuro-Immuno-Gastroenterology, Digestive Diseases Research Unit, Vall d’Hebron Institut de Recerca, Department of Gastroenterology, Hospital Universitario Vall d’Hebron, 08035 Barcelona, Spain; carmen.alonso@vhir.org (C.A.-C.); javier.santos@vhir.org (J.S.); 6 Centro de Investigación Biomédica en Enfermedades Hepáticas y Digestivas (CIBEREHD), 28029 Madrid, Spain

**Keywords:** intestinal barrier function, mucosal mast cells

## Abstract

The gastrointestinal tract harbours the largest population of mast cells in the body; this highly specialised leukocyte cell type is able to adapt its phenotype and function to the microenvironment in which it resides. Mast cells react to external and internal stimuli thanks to the variety of receptors they express, and carry out effector and regulatory tasks by means of the mediators of different natures they produce. Mast cells are fundamental elements of the intestinal barrier as they regulate epithelial function and integrity, modulate both innate and adaptive mucosal immunity, and maintain neuro-immune interactions, which are key to functioning of the gut. Disruption of the intestinal barrier is associated with increased passage of luminal antigens into the mucosa, which further facilitates mucosal mast cell activation, inflammatory responses, and altered mast cell–enteric nerve interaction. Despite intensive research showing gut dysfunction to be associated with increased intestinal permeability and mucosal mast cell activation, the specific mechanisms linking mast cell activity with altered intestinal barrier in human disease remain unclear. This review describes the role played by mast cells in control of the intestinal mucosal barrier and their contribution to digestive diseases.

## 1. Introduction 

Mast cells develop a fundamental defensive and immuno-regulatory function, particularly at the mucosal border between the body and the environment. The intestinal mucosa is the largest interface that separates the inner and outer environments constantly exposed to luminal content. It allows only small amounts of antigens and bacteria to cross the epithelium, while preventing the passage of potentially harmful substances. The ability to protect the body from harmful luminal content and control mucosal permeability constitutes the intestinal barrier function. This defensive function is highly regulated by immune and non-immune mechanisms, in which mast cells play a central role. Thanks to their great variety of receptors, mast cells respond to different types of stimuli, including microbial, neural, immune, hormonal, metabolic and chemical triggers. Mast cell response is vehiculised by the release of mediators contained in their cytoplasmic granules and lipid bodies or synthesised de novo [1], thereby exerting antimicrobial, neurological, immune and metabolic functions. Specifically, in the intestinal mucosa, mediators released by mast cells affect epithelial integrity and viability, promote ion and water secretion, stimulate innate and adaptive immune responses, blood flow, coagulation and vascular permeability, wound healing and fibrosis, and facilitate neuro-immune interactions which promote peristalsis and pain perception [2]. Normal functioning of the intestinal barrier is fundamental for homeostasis, while uncontrolled barrier mechanisms might lead to enhanced mucosal permeability and passage of luminal antigens and/or microorganisms across the intestinal epithelium, which potentially induce disturbances in epithelial–neuro-immune interactions that facilitate the development of inflammation in the gut. In fact, impaired epithelial barrier function has been largely implicated in the origin and development of many digestive and non-digestive diseases. Therefore, the tight regulation of intestinal permeability represents a central mechanism in the treatment and prevention of human disease.

Different methodological approaches have revealed an increased number of mast cells in the intestinal mucosa of patients with altered barrier function such as in inflammation-associated intestinal diseases and functional gastrointestinal disorders. Moreover, specific studies have shown a higher degree of activation of mucosal mast cells by means of the quantification of mast cell mediators and/or morphological analysis of the degranulation profile of cytoplasmic granules. Stabilising or blocking mast cell receptors provide, therefore, a promising tool to target disturbances in intestinal permeability and promote intestinal homeostasis. This review summarises the role of gastrointestinal mast cells in the regulation of intestinal barrier function and updates advances in the study of disease mechanisms associated with gastrointestinal diseases. 

## 2. Origin, Phenotype and Function of Gastrointestinal Mast Cells

Mast cells are long-lived granulated immune cells that reside in all vascularised tissues in the body. They derive from haematopoietic stem cells, which generate progenitor mast cells that circulate in low numbers in the blood and migrate to tissues in which they complete their differentiation process [2,3]. Their function, maturation and phenotype are the direct consequence of their interaction with the local microenvironment, including the production of a wide variety of membrane molecules involved in cell-to-cell or cell-to-extracellular matrix interaction [4], although pleiotropic, mast cells preferably reside in mucosal interfaces (skin, respiratory, genito-urinary and gut mucosa) in close contact with the environment, ready to react against infectious organisms, harmful substances and other environmental challenges. Intestinal homing of mast cells depends on the binding of α4β7 integrin with its corresponding adhesion molecules and the CXC chemokine receptor-2, both expressed in gastrointestinal mast cells [5]. Depending on the anatomical location, mast cells are categorised into connective tissue mast cells or mucosal mast cells. Based on their protease content, mast cells are classified as: mast cells containing high levels of tryptase but little or no chymase (MC_T_), mast cells containing chymase but little or no tryptase (MC_C_) and mast cells containing tryptase, chymase and carboxypeptidase (MC_TC_) [6]. However, mast cell classification based on protease content is ambiguous, since protease expression can change depending on the tissue environment.

Mast cells can be found in all layers within the gastrointestinal tract; however, the largest population resides in the lamina propria of the mucosa and in the submucosa. Specifically, MC_T_ are predominant in the intestinal mucosa (98%), while representing only 13% of all mast cells in the submucosa [7]. By contrast, MC_TC_ is the main phenotype in the intestinal submucosa, accounting for 77%, while the rest of the population displays an MC_T_ phenotype [8]. MC_C_ have also been identified but appear to be uncommon [6]. A new phenotype of mast cells expressing tryptase and carboxypeptidase A3, but not chymase, has recently been defined in the bronchial and oesophageal mucosa associated with the pathophysiology of asthma and eosinophilic oesophagitis, respectively [9,10]. However, exactly how this phenotype contributes to human disease remains unknown.

Mast cells are currently recognised as regulatory and effector cells in both innate and adaptive immunity. Their broad functions rely on their ability to react to a great variety of stimuli and secrete biologically-active products with pro-inflammatory, anti-inflammatory and/or immunosuppressive properties. Mast cells play a prominent role in immunoglobulin(Ig)-mediated allergic inflammation, and are also involved in a variety of intestinal and non-intestinal diseases such as gastrointestinal inflammation, functional gastrointestinal disorders, infections, autoimmune diseases, atherosclerosis and carcinogenesis [11] as well as in neuropsychiatric conditions [12]. Of importance for intestinal homeostasis, mast cells are fundamental for diverse intestinal physiological processes such as the regulation of mucosal integrity and epithelial barrier activity, and the maintenance of neuro-immuno interaction that supports the brain-gut axis. The fact that mast cells have persisted throughout vertebrate evolution, with an ancient origin even before the development of adaptive immunity, reinforces their importance in innate immunity as well as their remarkable role in such a variety of diseases [13].

## 3. Mast Cell Activation

The surface of mast cells is covered with a variety of receptors specific for immune ligands (Igs, complement fragments and cytokines) and for non-immune mediators, which include neurotransmitters, neuropeptides, hormones, growth factors and other biological and physicochemical stimuli [14]. Mast cell versatility implies they can be activated by different mechanisms, with cross-linking of IgE high-affinity receptor (FcεRI) to cell surface-bound IgE being the traditional and best studied stimulus in sensitised individuals [15]. A response is then triggered by a serie of phosphorylation cascades and activation motifs that lead to intracellular calcium flux, activation of transcription factors (such as AP-1, MITF and STAT-5), mast cell degranulation and cytokine production [16]. Mast cells can additionally be stimulated by IgG since they also express its receptor (FcγRI) [17] and other Ig-associated receptors. Of importance for the control of gastrointestinal disease is the recently identified IgG signalling via FcγRIIb, which suppresses a hypersensitivity reaction [18]. As innate immune sentinels, mast cells recognise microbial agents (bacterial, viral, parasitic and fungal) and endogenous factors derived from cell damage by germline-encoded pattern recognition receptors, which include toll-like receptors (TLRs), C-type lectin-like receptors (CLRs), retinoic acid-inducible gene I (RIG-I)-like receptors (RLRs) and nucleotide-binding oligomerisation domain (NOD)-like receptors (NLRs) [19]. Importantly for homeostasis and for ensuring an appropriate response to injury, mast cells also respond to different endogenous stimuli since they express receptors for neurotransmitters (such as acetylcholine and serotonin), neuropeptides (such as substance P, SP and vasoactive intestinal peptide, VIP), neurotrophins (such as nerve growth factor, NGF) and gaseous neurotransmitters (such as nitric oxide, NO). 

Upon activation, mast cells release biologically-active products (Table 1), newly synthesised or already contained in their cytoplasmic granules and lipid bodies [1]. The storage of these molecules in mast cell granules is possible, thanks to the anionic gel matrix composed of heparin and chondroitin sulphate, in which the mediators become trapped [20]. Pre-formed mediators include proteases, biogenic amines, proteoglycans, lysosomal enzymes, certain cytokines and growth factors and granule membrane-associated proteins. Newly-synthesised mediators include lipidic compounds, neuropeptides and a huge variety of cytokines, chemokines and growth factors [1,21]. This wide variety of molecules produced by mast cells supports their pleiotropic functions during both homeostasis and disease.

ADAMTS5, a disintegrin and metalloproteinase with thrombospondin motifs 5; TNF, tumour necrosis factor; IL, interleukin; GMCSF, granulocyte-macrophage colony-stimulating factor; bFGF, basic fibroblast growth factor; VEGF, vascular endothelial growth factor; NGF, nerve growth factor; VAMP, vesicle-associated membrane protein; MUNC, mammalian uncoordinated-18 protein; SCAMP, secretory carrier-associated membrane protein; LC3, lipidated light chain 3; MHC, major histocompatibility complex; CAP, cathelicidin antimicrobial peptide; SCF, stem cell factor; TGF, transforming growth factor; MCP, monocyte chemoattractant protein; RANTES, regulated upon activation normal T-cell expressed and secreted chemokine; TARC, thymus and activation-regulated chemokine; MCSF, macrophage colony–stimulating factor; PDGF, platelet-derived growth factor; GnRH, gonadotropin-releasing hormone. 

**Figure 1:**
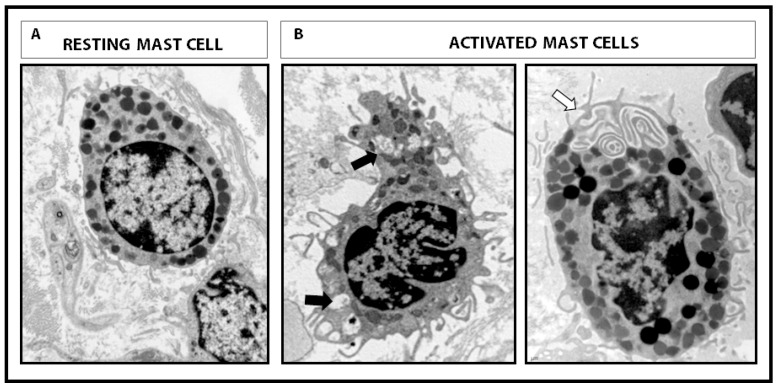
Intestinal mucosal mast cells. Representative micrographs of human mast cells showing different morphology at the ultrastructural level. (**A**) Resting mast cell with intact cytoplasmic granules and lipid bodies, displaying regular plasma cell membrane. (**B**) Activated mast cells showing piecemeal degranulation with loss of intra-granular electrodensity (black arrow) and inter-granular or granule-to-cell membrane fusion and typical channels (white arrow) during anaphylactic degranulation. Magnification 12,000×.

The secretion of mast cell mediators is carried out mainly by two mechanisms: piecemeal and anaphylactic degranulation (Figure 1). Piecemeal degranulation leads to partial or total granule emptying, causing a selective release of the content without inter-granule or granule-to-plasma membrane fusions. Ultrastructural analysis showed granule morphology to be quite conserved after the piecemeal process [22]. This type of secretion is promoted by neuropeptides, cytokines and microbial products that interact with mast cells, as described in physiological conditions and a variety of digestive diseases, among which inflammatory bowel disease (IBD), irritable bowel syndrome (IBS) and functional dyspepsia (FD) have been the most studied [23,24,25]. By contrast, anaphylactic degranulation is the explosive release of mast cell content by granule or granule-to-plasma membrane fusions followed by extrusion [26] and is associated with hypersensitivity reactions. Mast cell degranulation is mediated by the soluble N-ethylmaleimide-sensitive factor attachment protein receptors (SNARES), of which VAMP-7, VAMP-8, SNAP-23 and STX-4 have been reported to be significant SNARE molecules in human intestinal mast cell granule fusion and exocytosis [27,28]. The degree and type of activation of mast cells, as a consequence of their interaction with the microenvironment, determines the maintenance of homeostasis or disruption of essential defensive functions in the mucosa. This is of key importance for intestinal physiological activity, since the intestine represents the largest surface of the body in contact with the external environment and mast cells interact with virtually all cells in the mucosa as well as with microorganisms and harmful molecules that reach the intestine. Cell plasticity and responsiveness define mast cells as a fundamental component of intestinal barrier function during both homeostasis and disease.

## 4. Intestinal Barrier Function

The surface of the intestinal mucosa is lined with epithelial cells, which physically separate the intestinal lumen from the internal milieu, thereby avoiding the passage of potentially harmful substances while maintaining nutrient and electrolyte absorption. During evolution, the gut developed the intestinal barrier function, an effective defensive system involving intra-and extracellular elements, which closely interact to promote correct functioning of the epithelium, immune responses and acquisition of tolerance against food antigens and the intestinal microbiota. The loss of epithelial integrity facilitates antigen penetration into the mucosa, which triggers immunological responses which, in turn, increase epithelial permeability to luminal content, thereby promoting inflammation. An abnormal intestinal barrier has major implications for human health, being involved in the origin and development of many digestive (coeliac disease, IBD, IBS, food allergy) and extra-digestive diseases (schizophrenia, diabetes and sepsis, among others) [29,30]. The role of mast cells in intestinal barrier control is well documented; however, the specific mechanisms by which mast cells contribute to barrier dysfunction in human disease remain unclear. 

### 4.1. Elements of Intestinal Barrier Function

The intestinal barrier is composed of different elements, located in both the luminal and internal compartments. Residing in the lumen, the microbiota restricts pathogen colonisation and interacts with epithelial, immune and neural cells [31] to promote barrier function by mechanisms that include nutrient acquisition, energy and metabolism regulation [32,33], and cell proliferation [34]. Extracellular luminal elements include pH, enzymes from gastric, pancreatic and biliary secretions, the mucus layer and molecules released by epithelial and immune cells, which include defensins, lisozyme, phospholipids, trefoil factor family peptides, cathelicidins, ribonucleases and Igs, mainly secretory IgA [35,36]. Furthermore, peristaltism and water and chlorine secretion into the lumen wash out content and slow down antigen translocation to the lamina propria [37]. The epithelial layer is a fundamental and multifunctional element of the barrier responsible for barrier, digestive, metabolic and immune functions [37]. The epithelium includes specialised cells that produce and secrete mucus (goblet cells), defensins (Paneth cells), hormones and neuropeptides (enterochromaffin cells). It also includes a unique cell type specialised in antigen uptake from the lumen (M cells) [38]. To ensure an effective physical barrier, epithelial cells are tightly bonded to each other by intercellular junctions (tight junctions, TJ, at the apical junctional complex, followed by adherent junctions, and desmosomes). 

The immune contribution to the mucosal barrier is carried out by the gut-associated lymphoid tissue (GALT) distributed in organised lymphoid structures such as lymphoid follicles, Peyer’s patches and mesenteric lymph nodes, in which immune responses are initiated. GALT also includes a diffuse distribution of effector cells throughout the epithelium and the lamina propria of the intestinal mucosa [39], composed mainly of plasma cells, macrophages, mast cells, lymphocytes, eosinophils and dendritic cells [40]. Furthermore, connective tissue, blood and lymph vessels and fibroblasts that reside in the lamina propria also contribute to barrier function maintenance. An integrated network of neural cells from both the central and enteric nervous systems coordinates digestive functions and intestinal homeostasis maintenance via the release of neurotransmitters and, indirectly, neuro-immune interactions.

### 4.2. Mast Cells as Neuro-Immune Players in the Regulation of Intestinal Barrier Function 

Control of the intestinal barrier results from a network of interactions among the microbiota, epithelial cells and immune and nervous systems. The communication between the central and enteric nervous systems, the so-called brain-gut axis, permits regulation of the intestinal barrier by monitoring ion secretion, epithelial tightness, immune function and peristalsis. The functional unit established by mast cell-nerve interaction [41] is a fundamental component in such interplay via paracrine signalling (the most common), transgranulation (granule fragments and mediators are delivered directly to the neuronal body) and integrin signalling (physical synapses through integrins) [21]. Enteric neurons, as well as vagal and spinal afferents, express receptors for molecules (mainly proteases, neuropeptides, hormones and growth factors) released by mast cells, which stimulate nerve terminals, thereby modulating the firing threshold. Similarly, neuropeptides and neurotransmitters released by neurons stimulate mast cell secretion of mediators, which further activate neuronal receptors [21], supporting the maintenance of this neuro-immune interplay. This interaction contributes to monitoring of gut function by the central nervous system; however, if overstimulated, it can exert harmful effects associated with disease [42]. 

The contribution of mast cells to barrier function through neuro-immune mechanisms has been evidenced in different experimental settings. Diverse stressors (physical and psychological, acute and chronic) have been shown to disturb barrier homeostasis by increasing ion secretion and epithelial permeability [43,44], effects avoided in mast cell knock-out rats and also in humans after oral pretreatment with a mast cell stabiliser [45,46]. The stress response, which includes endocrine and behavioural changes, is centrally mediated by the release of corticotropin-releasing factor (CRF). Additionally, intestinal mucosal cells, including immunocytes, nerves and enterochromaffin cells, produce and release CRF upon activation. Local CRF interacts with its receptors (CRF-R1, CRF-R2) on subepithelial mast cells [47] to induce mucin release, ion and water secretion, and increase epithelial permeability [46,48,49]. Moreover, other neuropeptides such as SP and NGF induce the release of vasoactive mediators from mast cells, thereby contributing to chloride secretion, barrier dysfunction, hyperalgesia, diarrhoea, inflammation and motility changes [50]. In fact, epithelial permeability to luminal bacteria seems to be modulated by VIP and mast cell activity, as suggested by the VIP–mast cell-dependent regulation of commensal and pathogenic bacteria passage in the human colon [51].

## 5. Regulation of Intestinal Mucosal Barrier by Mast Cells 

**Figure 2:**
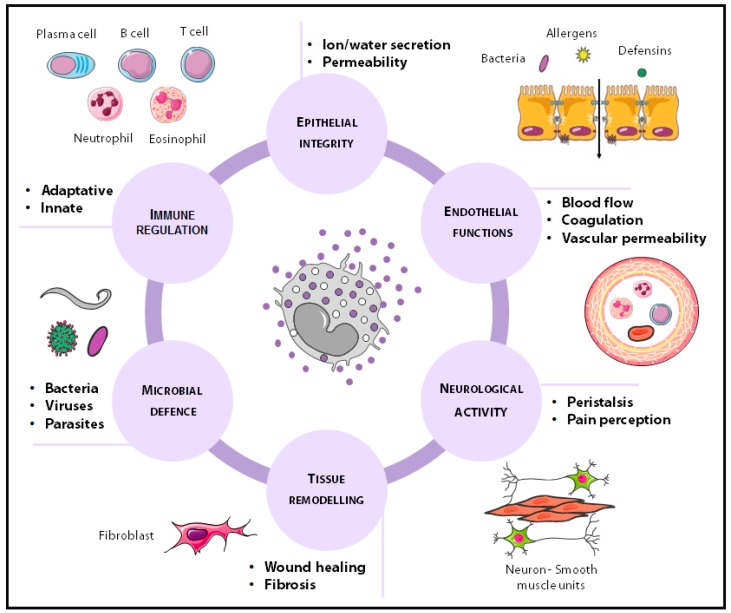
Physiological functions of mast cells in the gastrointestinal tract. Mucosal mast cells play an important role in multiple functions necessary for gut homeostasis, including epithelial, endothelial and neurological functions, tissue transformation, host defence, and immunity.

Mast cells are unique due to their ability to modulate their phenotype, a process called transdifferentiation [52] by which they synthesise and release a specific mediator profile depending on the microenvironment [53]. The anatomical complexity of the intestine and the ever-changing environment contribute to mast cell phenotype which, based on the nature of mediators released into the extracellular milieu, determines organ function. In this way, intestinal mast cells perform multiple functions necessary for homeostasis, including the regulation of epithelial activity (ion and water secretion and permeability), endothelial functions (blood flow, coagulation and vascular permeability), tissue transformation (wound healing and fibrosis), neurological functions (neuro-immune interactions, peristalsis and pain perception), host defence (bacterial, viral and parasitic infections) and innate and adaptive immunity [2] (Figure 2). The most prominent contributions of mast cells to mucosal barrier control are mediated through mechanisms that modulate epithelial function and innate and adaptive defensive responses.

### 5.1. Regulation of Epithelial Function

Among the large variety of molecules released by mast cells, evidence of their contribution to epithelial integrity stems from studies designed to reveal the role of proteases, histamine and cytokines (Table 2). 

#### 5.1.1. Tryptase 

This enzyme is the most abundant secretory granule-derived serine proteinase contained in mast cells and has been largely implicated in epithelial permeability. It activates a protease-activated receptor (PAR2) [54], expressed on both the apical and basolateral membranes of intestinal epithelial cells, promoting TJ disruption and increasing intestinal permeability [55]. Tryptase cleavage and activation of PAR2 lead to calcium mobilisation, beta-arrestin and ERK1/2 MAPK activity, perijunctional F-actin redistribution [56,57], Zonula-1 (ZO-1) delocalisation [58] and protein junctional adhesion molecule-A (JAM-A) downregulation [59]. Tryptase is also able to damage cells, as interpreted by a reduction in cell viability together with an increase in lactate dehydrogenase activity and apoptosis [57]. 

#### 5.1.2. Chymase 

This chymotrypsin-type serine protease is implicated mainly in extracelluar matrix degradation and also targets epithelial integrity through activation of the PAR2 receptor. The biological function of chymase has been studied through the generation of mouse strains deficient in different murine mast cell proteases (mMCP), functional homologues to human chymase: mMCP-1 mMCP-2 mMCP-4 or mMCP-5 [60]. Experimental studies have shown that the activation of PAR2 receptors by chymase induces p38 phosphorylation and p44/42 (ERK1/2) signalling pathway activation, which increase metalloprotease-2 (MMP-2) and reduce claudin-5, resulting in epithelial barrier dysfunction [61]. Specifically, the rat mucosal mast cell chymase, rMCP-2, increases epithelial permeability through alteration of ZO-1 and occludin distribution in epithelial cells [62] and cleavage of occludin, cadherin 17 and protocadherin alpha [63]. 

#### 5.1.3. Histamine 

This amine is a preformed mast cell mediator involved in a variety of physiological and pathological processes throughout the gastrointestinal tract. It mediates immunological responses, visceral nociception, modulation of intestinal motility and gastric acid secretion through activation of its receptors (H1-H4) [64]. The contribution of histamine to epithelial dysfunction is mediated by H1 receptors directly stimulating chloride secretion, as demonstrated in the human colonic epithelium [65]. Recent evidence also revealed a role of histamine in increasing epithelial intestinal permeability and bacterial translocation in malaria-infected mice [66]; however, the specific mechanisms leading to barrier deregulation remain unknown.

#### 5.1.4. Cytokines 

These small molecules are released mainly by lymphocytes, macrophages, eosinophils, dendritic cells and mast cells that mediate inter-cell communication. Mast cells produce a large variety of cytokines, many of which have a direct impact on the intestinal epithelial barrier (Table 2). TNF-α directly disrupts TJ via myosin light-chain kinase (MLCK)-mediated phosphorylation of the myosin light chain (MLC) [67,68] and ZO-1 and occludin downregulation [68,69]. Interleukin (IL)-4 stimulates mast cell IL-13 production [70], with both molecules sharing the IL-4Rα chain receptor, which elicits phosphatidylinositol 3-kinase (PI3K) activation and modulation of epithelial paracellular permeability [71]. IFN-γ also alters paracellular permeability in the intestine through the reduction of claudin 2 and 3 and reorganisation of claudin 4 [72]. IL-1β regulates intestinal function mainly via activation of the NF-κB pathway and the MLCK gene [73,74,75]. IL-9 also increases intestinal permeability associated with a genetic profile identified in intestinal anaphylaxis [76], and IL-6 promotes JNK activation of AP-1 and upregulation of the claudin 2 gene, leading to TJ disruption [77]. On the other hand, the anti-inflammatory cytokine IL-10 has also been shown to develop a protective role in intestinal barrier function, since IL-10-gene-deficient mice showed increased intestinal permeability [78,79] and the administration of IL-10 prevented IFN-γ-induced barrier dysfunction [80]. However, IL-10 has been shown to enhance IgE-mediated mast cell activity, which suggests a potential contribution to barrier dysfunction during food allergy response [81]. 

### 5.2. Regulation of Mucosal Immunity

A multivalent capacity to recognise and respond to both internal and external dangers, together with the ability to cross-talk with other immune cells, render mast cells a unique player in linking innate and adaptive immunity. 

#### 5.2.1. Mast Cells in Innate Immune Responses

Innate immunity constitutes the first line of defence against invading pathogens and performs an essential task prior to the development of adaptive immunity. Mast cells play a protective role against bacterial and viral infections via mechanisms that include the recruitment of neutrophils, eosinophils and macrophages to the mucosa [82] and the activation of defensive responses (Table 3). Mast cells release phospholipase A2 and extracellular enzymes with proinflammatory and antibacterial activities [83]. These enzymes exert a direct killing effect on pathogens through mast cell extracellular traps (MCET), which consist of extracellular extensions/fibres composed of DNA, histones, proteases, tryptases and anti-microbial peptides. MCET are actively formed by mast cells when they are not able to exert efficient phagocytosis of clumped bacteria. MCET formation relies on the production of reactive oxygen species and culminates in the death of mast cells [84]. MCET formation seems to be induced by hypoxic stress, thanks to the hypoxia-inducible factor 1α (HIF-1α) [85], but also by the increase in cytokines such as IL-23 and IL-1β [86]. Specific bacterial proteins such as Streptococcus pyogenes M1 protein also appear to activate this mast cell response [87]. A further mechanism by which mast cells participate in innate immunity is phagocytosis. The phagocytic activity displayed by mast cells against some bacteria (e.g., Klebsiella Pneumoniae, Escherichia Coli, Streptococcus faecium, Citrobacter freundii) [88] could be mediated by different mechanisms: through the surface complement receptors that detect and opsonise bacteria [89] or the recognition of bacterial Fim-H adhesins, via CD48 receptors [90]. Antimicrobial peptides released by mast cells include cathelicidins, which also act as leukocyte chemoattractants through interaction with the Formyl peptide receptor–like 1 (FPRL1) of leukocytes [84], leading to degranulation of β-tryptase [91] which, in turn, degrades cathelicidin [92]. This suggests that mast cells exert an auto-regulatory autocrine regulation that permits immune modulation through a negative feedback loop. Mast cell proteases participate in innate immunity by different mechanisms. Tryptase seems to be essential to fight bacterial infection through the recruitment of neutrophils [93]. Chymase contributes to inhibiting Streptococcus attachment to the extracellular matrix via the proteolytic degradation of fibronectin [94] and plays a role in promoting parasite expulsion [95]. Carboxypeptidase A favours the clearance of endogenous and exogenous toxins [96,97], and histamine released by mast cells initiates neutrophil infiltration into the colonic mucosa through H4R, as demonstrated in a murine model of experimental colitis [98]. 

#### 5.2.2. Mast Cells in Adaptive Immune Responses

Adaptive immunity induces specific and memory responses to certain antigens. Mast cells support T-cell and B-cell activation and provide fine-tuning of tolerance and immune suppression (Table 4). 

Interaction with T lymphocytes: mast cells promote activation, recruitment, proliferation and cytokine secretion in multiple T-cell subsets, although the most well-known interaction is their involvement in Th2-mediated inflammation associated with allergic disease [99]. Moreover, close physical proximity between mast cells and T cells has been demonstrated in several T cell-mediated inflammatory processes. Mast cell have been shown to be activated by T cells, an effect that is mediated by T cell -derived microvesicles and results in specific proinflammatory cytokine release [100,101]. Mast cells release a wide variety of chemotactic factors for different CD4^+^ T-helper (Th) cell subsets: CCL3, CCL4, CXCL9 and CXCL10 for Th1; CCL5 and CCL11 for Th2; and CCL2 and CCL20 for Th17 [102]. Furthermore, mast cells also support the polarisation of Th cell responses through the secretion of specific mediators such as IL-12 and IFNγ for Th1; IL-4 for Th2; IL-6 and TGFβ1 for Th17; and IL-6 and TNFα for Th22 [103]. Thanks to their pathogen-recognising receptors, mast cells internalise and process antigens that are presented to T cells by MHC class II mechanisms [104]. Furthermore, after activation, mast cells secrete TNFα, which binds TNFRI and TNFRII on T cells to regulate T-cell activation, surface expression of OX40, ICOS, PD-1 and other co-stimulatory molecules on CD4^+^ cells, thereby enhancing T-cell proliferation and cytokine secretion [105]. In addition, mast cells seem to downregulate the suppressive function of T regulatory cells (Treg) by IL-6, as well as establishing a direct contact through OX40L on mast cell membranes and the receptor OX40 on the Treg surface receptor [106]. Histamine released by mast cells also regulates Treg cells; thus, interaction with H1 receptors in Tregs induces a reduction in CD25 and Foxp3 expression, leading to a decrease in Treg suppressive activity [107]. 

Interaction with B lymphocytes: mast cells directly support IgE production through the secretion of IL-4 and IL-13 and the interaction between CD40L and CD40 expressed on mast cells and B cells, respectively [108]. Moreover, mast cells are also involved in B cell proliferation and differentiation into IgA-secreting plasma cells by direct interaction (CD40/CD40L) and the secretion of IL-6, IL-5 and transforming growth factor-β (TGF-β) [109]. Other cytokines released by mast cells, IL-5 and IL-33, are involved in the production of IgM and play a crucial role in tissue homeostasis and defence against mucosal pathogens [110]. B lymphocyte survival and proliferation are also influenced by mast cells through the secretion of cytokines and chemokines, also at distal sites through the release of exosomes, which contain proteins, RNA, soluble mediators, FcεRI receptors, MHC class II proteins and co-stimulatory molecules [111]. Exosomes are released and bind to receptors on target cells such as B cells. Once internalised, messages delivered by exosomes promote the generation of IL-2, IL12, IFNγ, IgG1 and IgG2 by B cells [112]. 

## 6. Experimental Procedures to Evaluate Intestinal Mast Cells

**Figure 3:**
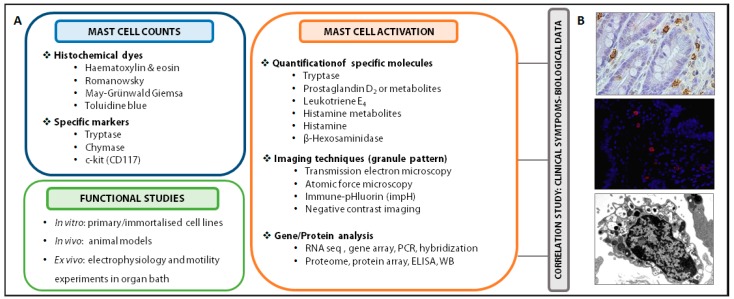
Experimental procedures to evaluate mast cells. (**A**) Experimental procedures to evaluate mast cell counts, activation and functional studies. Identification and counting of mast cells can be performed by histochemical dyes and specific staining (above left); activation of mast cells can be assessed by quantification of specific molecules, imaging techniques (granule pattern) and gene and/or protein expression analysis of specific mediators or genes (right); functional studies in in vitro experiments (primary or immortalised cells), in vivo models (mice, rats) and ex vivo by means of organ bath experiments for evaluating mast cell-dependent changes in barrier function or muscle contractility. In addition, a correlation study to associate clinical symptoms with biological data can be conducted. (**B**) Representative images of intestinal mucosal mast cells: tryptase staining with immunohistochemistry (top, 400×) and immunofluorescence (middle, 400×) and mast cell displaying piecemeal degranulation, observed by electron microscopy (botton, 15,000×). ELISA, enzyme-linked immunosorbent assay; WB, western blot.

Mucosal mast cell infiltration and activation can be assessed by several methods (Figure 3). Morphological analysis in tissue specimens is recommended for identifying cell architecture and mast cell location through simple histochemical dyes (with toluidine blue being the most specific) or by using antibodies anti-mast cell proteins via immunohistochemistry or immunofluorescence techniques (tryptase, chymase, c-kit, FcεR1) [113,114]. Mast cell activation can be assessed by analytical methods quantifying mast cell mediators or metabolites in different biological samples (urine, blood, luminal content, biopsy, cell suspension, cell culture supernatant). For example, tryptase content in the intestinal lumen as well as tryptase gene expression in mucosal biopsies have been identified in activated mucosal mast cells in IBS patients and associated with symptom severity [115,116]. Urinary prostaglandin D_2_ and leukotriene E_4_ levels are also raised after mast cell activation, although the use of the former as a single marker is not recommended and the latter has not been fully validated. Urinary histamine has been widely used as a specific marker; however, its metabolites are influenced by diet or bacterial contamination and its concentration in blood can also derive from basophils and be affected by several factors such as blood sample handling [117]. Since histochemical staining does not provide information on granule secretion at subcellular level, ultrastructural analysis is necessary to establish the type and degree of degranulation. Different imaging techniques are available, with electron microscopy being the most commonly used, since high magnification reveals cellular and subcellular structure. Transmission electron microscopy allows us to determine whether mast cells display a secretory profile, type of degranulation, granular and plasma cell membrane morphology and the proximity to other cells or structures within the tissue. Nevertheless, it has some limitations since the dehydration of samples or imaging under vacuum can affect cell membranes. A complementary technique is atomic force microscopy, which preserves structural integrity and permits cell analysis of high-resolution 3D-generated images. Degranulation of mast cells can be studied in vivo using a fluorescent protein-based indicator of degranulation named immune-pHluorin (impH) that identifies changes in fluorescence according to the pH value. The study of secretory granule biogenesis, maintenance, regulation and recycling can also be analysed using negative contrast imaging [113]. Gene and protein expression (after sample fixation for RNA/protein stabilisation) has been analysed to identify a mast cell-associated gene/protein profile or to assess the expression of specific molecules. 

Functional assays can also be performed in different experimental settings to reveal activation/inhibition mechanisms of mast cells. Different immortalised mast cell lines (LAD-2, HMC-1, LUVA) or isolated primary cells (blood progenitors or tissue mast cells) are currently being used individually or in co-culture with other leukocytes or intestinal epithelial cell lines. Besides, in vitro mast cell degranulation can be detected and quantified by measuring histamine or β-hexosaminidase release to culture medium [118]. On the other hand, in vivo studies using animal models such as IL-9 transgenic mice [76], Cre/loxP mast cell-deficient mice [119] or mast cell knock-out rats [45] can also reveal the role of mast cells in the intestinal mucosa. Furthermore, mast cell-dependent changes in barrier function or motility can be evaluated by electrophysiological measurements in ex vivo tissue specimens mounted in Ussing chambers or in an organ bath for the assessment of muscle contractility. In this setting, a pharmacological approach can be designed in which activation/blockade can help to reveal the effect of mast cells on molecule transport across the epithelial barrier or muscle contractility. Finally, a correlation study is essential for linking clinical manifestations, digestive dysfunction and mast cell activation [120].

## 7. The Role of Mast Cells in Digestive Disease

### 7.1. Food Allergy

Mast cells are the main effector participant in allergic responses as a result of a series of interactions among T cells, B cells, antigen-presenting cells and IgE production. Food allergy can be classified, depending on the nature of the immune response, as IgE-mediated, non-IgE-mediated or mixed [121], with mast cells being implicated in both types of response. Patients with food allergy display a high concentration of mast cell mediators such as histamine, TNF-α, IL-5 and tryptase in serum, urine, gut lavage fluid, stool and intestinal biopsies [122,123]. As mucosal mast cell activation increases intestinal permeability, mast cells can also contribute to the initiation of food allergic inflammation through a greater influx of allergens and microbes to the lamina propria. In fact, the ion secretion associated with intestinal anaphylaxis has been mainly attributed to mast cells [124]. Furthermore, the involvement of neuro-immune interactions has been reported in allergic disease [125,126], demonstrating that neurons communicate and regulate mast cell activation during allergic inflammation and, in turn, mast cells, reciprocally, stimulate nerve endings. Mast cells are involved in gastrointestinal and systemic manifestations of food allergy. However, systemic anaphylaxis has been linked only with connective tissue mast cells, and gastrointestinal food allergy has been related to both connective tissue and mucosal mast cells. Thus, although it seems clear that mast cells are essential effectors in food allergy, it is not fully known why allergen exposure can trigger these different clinical manifestations, thereby underlining mast cell heterogeneity as an essential contributor to these differences [127]. Nevertheless, little is known of the mechanisms leading to sensitization to food allergens. IL-9 transgenic mice showed that IL-9-mediated mast cell responses play an important role in food allergy and oral antigen sensitisation [76]. Therefore, apart from a pro-inflammatory role, mast cells also modulate allergic sensitisation and downregulate allergic inflammation [128], dual roles that impact on allergic responses and warrant further study.

### 7.2. Inflammatory Bowel Disease

IBD includes two main entities, ulcerative colitis (UC) and Crohn’s disease (CD), in which chronic gut inflammation results from altered host-microbial interactions in genetically-susceptible individuals [129]. The contribution of mast cells to IBD has been demonstrated in human and experimental studies [130] in which increased numbers of mast cells were found in tissue specimens from both UC and CD patients [131,132,133], showing ultrastructural changes with evidence of piecemeal and anaphylactic degranulation. Different types of mast cell mediators involved in IBD pathogenesis include TNF-α, IL-6, SP, histamine, prostaglandins and leukotrienes [134,135,136,137]. Altered brain-gut interactions have also been detected in IBD [138] and mast cells are thought to play a part in the neural inflammation present in these patients [139]. In fact, in the DSS experimental model of colitis, the number of mucosal mast cells in close proximity to VIP nerves was significantly increased [140]. Moreover, alterations in intestinal ion transport [141], the fibrotic response in CD [142,143], microbiota dysbiosis [144], fibroblast proliferation, collagen production and contractile activity have also been associated with intestinal mast cell activation [145]. The participation of mast cells in IBD is undeniable, since they play a role in several aspects of the disease, among which intestinal permeability, initiation and maintenance of inflammatory processes (with ensuing tissue remodelling) and transmittance of signals during neuropathological stress [146] are noteworthy. Therefore, mast cell-stabilising drugs or drugs interfering with mast cell mediators are considered an additional therapeutic possibility in the treatment of IBD [147].

### 7.3. Coeliac Disease

Coeliac disease is a chronic inflammatory disorder in the small intestine caused by intolerance to gluten. It implies remodelling of the intestinal mucosa where immune cells accumulate as a consequence of both adaptive and innate immune responses to undigested gliadin peptides [148]. A relationship between mast cells and coeliac disease has been reported, showing increased numbers of mast cells and their mediator histamine in the small intestine [149]. Moreover, the jejunum of coeliac disease patients shows inflammation caused by histamine, together with albumin secretion, resulting from endothelial disruption, which facilitates mucosal leakage [150]. Mast cells have been found to directly react to gliadin fragments by releasing proinflammatory mediators, and have been associated with increased neutrophil accumulation, prevalence of M1 macrophages, and severity of tissue damage during onset and progression of the disease [151]. Hence, mucosal mast cell count has been suggested as a marker for monitoring coeliac disease severity and a target for re-establishing gut tolerance to gluten.

### 7.4. Irritable Bowel Syndrome

IBS is a functional gastrointestinal disorder for which, despite intensive research, no biomarker has been identified to date. In the intestinal mucosa of IBS patients, a low-grade inflammatory infiltrate, characterised by an increased number of mucosal mast cells and T lymphocytes, has been reported in all clinical IBS subtypes in the small and large intestine [152]; however, not all studies reported similar results [153]. Despite disparities in immune cell counts among studies, altered intestinal barrier with increased epithelial permeability and disruption of TJ appeared to be a common finding [154]. The loss of functional integrity may facilitate the uncontrolled flux of antigens (food, microorganisms, toxins, etc) across the epithelium and stimulation of immunological responses in the lamina propria [155], thereby further increasing paracellular epithelial permeability and promoting low-grade mucosal inflammation. As tryptase is implicated in intestinal barrier deregulation, the generation of gastrointestinal motor abnormalities and visceral pain [116,156], mast cell activation may be of greater importance than number, as tryptase production, and not cell counts, correlates with TJ disruption and clinical symptoms, at least in the small intestine of IBS-D [116]. Moreover, the number of colonic mucosal mast cells in proximity to nerves positively correlates with abdominal pain severity in IBS [157]. Since mast cells form a link between the brain and the gut by local neuro-immune interaction, they mediate mucosal responses to central stimuli such as psychological stress, thanks to their location near nerve fibres and the presence of receptors for CRF or SP. Remarkably, the high prevalence of psychiatric comorbidities in patients with gastrointestinal disorders [158] highlights the significance of stress in the aetiopathogenesis of IBS. On these lines, recent research revealed associations between immune activation (humoral activity) and psychiatric comorbidities [159] and between stress episodes and the initiation/exacerbation of functional gastrointestinal disorders [160]. Evidence of mast cell implication in IBS pathophysiology is also supported by studies identifying an improvement in gut symptoms after administration of the stabiliser disodium chromoglycate [24,161,162] or ketotifen, a histamine H1-receptor antagonist and mast cell stabiliser, which led to reduced visceral perception, particularly in hypersensitive IBS patients [163].

### 7.5. Functional Dyspepsia

FD, one of the most common functional gastrointestinal disorders, is characterised by a diversity of symptoms occurring in the epigastric region. As with IBS, no biomarker has been identified, despite an increased number of mast cells and eosinphils being observed in the duodenal [164] and gastric [165,166] mucosa together with epithelial barrier dysfunction. Moreover, a recent meta-analysis confirmed this previous evidence [167]. Notably, in FD, there is a higher number of mucosal mast cells with an activated phenotype; however, this finding does not appear to correlate with the impaired barrier integrity observed in duodenal mucosa [25]. Despite these results, evidence still suggests that mast cell activation may play a role in the pathophysiology of FD, since granule morphology significantly differed when FD and control mucosal mast cells were compared, suggesting a differential synthesis and storage of mediators in mast cell granules [25]. Therefore, further studies are required to elucidate the role of mast cells in this disorder.

### 7.6. Mast Cell Activation Disorder

Mast cell activation disorders cover a wide range of entities, from relatively common IgE-mediated disease and chronic urticaria to rarer conditions such as mastocytosis or monoclonal mast cell activation disorder. Patients with symptoms stemming from a mast cell activation disorder, which do not meet the criteria for anaphylaxis, are considered for the diagnosis of mast cell activation syndrome, a condition for which gastrointestinal symptoms are well documented [168]. In fact, the symptoms have been considered as secondary to mast cell infiltration of the gut, in addition to deregulation of the local release of mast cell mediators such as histamine, prostaglandin, gastrin, SP and VIP [169,170,171,172,173,174]. The most common symptoms are abdominal pain, nausea/vomiting, diarrhoea, gastrointestinal bleeding and visceromegaly [174]. However, it remains unclear whether these symptoms arise from locally activated mast cells or mediators derived from sites distant from the gastrointestinal tract. Further studies are needed to improve understanding in this field.

## 8. Concluding Remarks

In summary, mucosal mast cells contribute to homeostasis and are actively involved in a variety of gastrointestinal diseases. Considering the heterogeneity of digestive entities to which mast cells contribute, it is indisputable that mast cells are able to influence and regulate gastrointestinal function through different mechanisms. In this context, the role mast cells play in epithelial barrier function maintenance, neuro-immune interaction and the regulation of mucosal immunity is remarkable. Therefore, considering the available data, future therapy approaches to stabilising mast cells constitute a promising tool for the improvement in gastrointestinal disorders associated with altered barrier function.

## Figures and Tables

**Table 1:** Mast cell mediators.

Pre-Formed Mediators	
**Proteases**	Mast cell-specific: tryptase, chymase, carboxypeptidase A Non-mast cell-specific: cathepsin G, granzyme B, active caspase 3, ADAMTS5, renin
**Biogenic amines**	Histamine, serotonin, dopamine, polyamines
**Proteoglycans**	Serglycin, chondroitin sulphates, heparin
**Lysosomal enzymes**	β-hexosaminidase, β-glucuronidase, arylsulphatase, cathepsins
**Cytokines/growth factors**	TNF, IL-4, GMCSF, bFGF, VEGF, NGF
**Granule membrane-associated proteins**	VAMPs, syntaxin 3, synaptotagmins, MUNCs, SCAMPs, CD63, RABs, LC3-II, MHC class II
**Others**	Heparanase, CAP-18, secretogranin-III and chromogranin A
**Newly-Synthesised Mediators**	
**Lipid mediators**	Leukotriene C4/B4, prostaglandin D2, platelet-activating factor
**Cytokines**	IL-1, IL-3, IL-6, IL-18, TNF, SCF, TGF-β
**Chemokines**	MCP-1, RANTES, eotaxin, TARC
**Growth factors**	GMCSF, MCSF, bFGF, PDGF, NGF, VEGF, GnRH

**Table 2:** Mast cell mediators and mechanisms associated with epithelial barrier dysfunction.

PROTEASES			
	Sample	Effect/ Implicated Mechanism	Ref.
**Tryptase**	**Increased intestinal permeability**		
	T84 intestinal epithelial cell line	Activation of PAR2 via ERK1/2 MAPK Reorganisation of perijunctional F-actin	[56]
	IEC-6 rat intestinal epithelial cell line	Activation of PAR2 via ERK MAPK	[57]
	MDCK epithelial cell line	Activation of PAR2 via p38-MAPK activation Disruption of tight junctions, relocalisation of ZO-1	[58]
	IBS caecum biopsies	Reduced JAM-A expression	[59]
**Chymase**	**Increased intestinal permeability**		
	Caco-2 intestinal epithelial cell line	Activation of MMP-2 signalling through PAR2 Reduction in CLDN-5	[61]
	MDCK epithelial cell line	Effect on the paracellular route Altered distribution of ZO-1 and OCLN	[62]
	Phage display analysis for rMCP-2 cleavage specificity	rMCP-2 cleaves OCLN, cadherin 17 and protocadherin alpha 4	[63]
**HISTAMINE**			
	Human colonic epithelium	Epithelial dysfunction/ Stimulates chloride secretion	[65]
	Antihistamine treatment in mice with malaria	Reduced gut permeability and bacterial translocation	[66]
**CYTOKINES**			
	**Increased intestinal permeability**		
**TNF-α**	Caco-2 intestinal epithelial cell line	Opening of the intestinal barrier TJ NF-kB p50/p65 binding and activation of the MLCK promoter	[67]
	Caco-2 and T84 intestinal epithelial cell line	Transepithelial resistance decreased MLC phosphorylation promotes TJ disruption Decreased expression of ZO-1 and OCLN	[68]
	Human intestinal cell lines HT-29/B6	Downregulation of OCLN	[69]
**IL-4 and IL-13**	T84 intestinal epithelial cell line	Decreased transepithelial resistance via PI3K pathway	[71]
**IFN γ**	T84 intestinal epithelial cell line	Decreases in CLDN-2 and 3 Redistribution of CLDN-4	[72]
**IL-1β**	Caco-2 intestinal epithelial cell line	Activation of NF-kB pathways	[73]
	Caco-2 intestinal epithelial cell line	Activation of the NF-κB pathway and the MLCK gene	[74]
	Human corneal epithelial cells	Loss of corneal epithelial barrier function Dependent on NF-κB Redistribution of ZO-1 and OCLN	[75]
**IL-9**	IL-9 overexpression in mice	Decrease in transepithelial electrical resistance Increase in jejunal permeability to FITC-dextran and HRP	[76]
**IL-6**	Caco-2 intestinal epithelial cell line Mouse intestinal perfusion	JNK activation of AP-1 and upregulation of CLDN-2-gene	[77]
	**Protective role in intestinal barrier function**		
**IL-10**	IL-10 gene-deficient mice	Increased intestinal permeability	[78], [79]
	Human endothelial solute barrier	Blockage of IFNγ-induced epithelial permeability	[80]

PAR2, protease-activated receptor-2; ERK, extracellular signal-regulated kinase; MAPK, mitogen-activated protein kinase; MDCK, Madin-Darby canine kidney; ZO-1, zonula occludens-1; IBS, irritable bowel syndrome; JAM-A, junctional adhesion molecule-A; MMP-2, matrix metalloproteinase-2; CLDN, claudin; OCLN, occludin; rMCP, rat mast cell protease; TJ, tight junctions; NF-kB, nuclear factor-kappa B; MLCK, myosin light-chain kinase; MLC, myosin light chain; PI3K, phosphoinositide 3-kinase; IL, interleukin; HRP, horseradish peroxidase; FITC, fluorescein; JNK, c-Jun N-terminal kinase; AP, activator protein; IFN, interferon.

**Table 3:** Mast cell implication in innate immunity.

Mast Cell Mediators	Target Cell/Molecule	Effect/ Implicated Mechanism		Ref.
**TNFα**	Neutrophils Eosinophils Macrophages	Recruitment of innate immune cells Proinflammatory effect		[82]
**sPLA2**	Eosinophils Macrophages	Activation of innate immune cells Proinflammatory effect		[83]
**MCTES:** Extracellular fibres composed of DNA, histones, proteases and AMP	Bacteria	Antibacterial effect		[84]
**Complement receptors**	Complement attached to bacteria	Phagocytosis Antibacterial effect		[89]
**CD48**	Bacterial adhesins (Fim-H)			[88] [90]
**AMP:** Cathelicidins	Pathogens	Antibacterial effect		[84]
**Tryptase**	Neutrophil	Recruitment	Pro-inflammatory effect	[93]
**Chymase**	Fibronectin	Extracellular matrix degradation	Inhibition of Streptococcus attachment to EM	[94]
**Carboxypeptidase**	Exogenous toxins	Proteolysis	Protective effect	[96], [97]
	Endogenous toxins	Proteolysis	Homeostatic effect	
**Histamine**	Neutrophil	Recruitment	Pro-inflammatory effect	[98]

TNFα, tumour necrosis factor alpha; sPLA2, phospholipase A2; MCTES, mast cell extracellular traps, DNA, deoxyribonucleic acid; AMPS, antimicrobial peptides; Fim-H, type 1 fimbrin D-mannose-specific adhesion; EM, extracellular matrix.

**Table 4:** Mast cell implication in adaptive immunity.

Mast Cell Mediators	Target Cell	Effect/Implicated Mechanism	Ref.
CCL3, CCL4, CXCL9, CXCL10	Th1	Adaptative immune cell recruitment Pro-inflammatory effect	[102]
CCL5, CCL11	Th2		
CCL2, CCL20	Th17		
IL-12, IFNγ	Th1	Polarisation of Th responses	[103]
IL-4	Th2		
IL-6, TGFβ1	Th17		
TNFα	Th22		
MHC class I and II	T cells	Ag presentation Pro-inflammatory effect	[104] [105]
TNFα	T cells	Activation Proliferation	[105]
IL-6	Receptors on Treg cells	Inhibition Pro-inflammatory effect	[106]
OX40L	OX40		
Histamine	H1 receptors	Decrease in CD25 expression and Foxp3 transcription in Treg cells	[107]
IL-4, IL-13	B cells	Class switch recombination into IgE producing plasma cells	[108]
IL-6, IL-5, TGFβ	B cells	Class switch recombination and differentiation into IgA producing plasma cells	[109]
CD40L	CD40 receptor on B cells	Co-stimulatory signal for Ig class switching	[108,109]
IL-5, IL-33	B cells subtype B-1	Stimulation IgM production. Participation in homeostasis and pathogen defence response	[110]
Exosomes containing: RNAs, soluble mediators, FcεRI, MHC II	Receptors on B cells	Promotion of IL-2, IL-12, IFNγ, IgG1, and IgG2 synthesis	[111]

CCL, chemokine (C-C motif) ligand; CXCL, chemokine (C-X-C motif) ligand; Th, T helper; IL, interleukin; IFNγ, interferon gamma; TGFβ1, transforming growth factor beta 1; TNFα, tumour necrosis factor alpha; MHC, major histocompatibility complex; Ag, antigen; Tregs, T regulatory cells; OX40L, OX40 ligand; H1, histamine receptor type-1; FOXp3, forkhead box P3; CD40L, CD40 ligand; Ig, immunoglobulin; RNA, ribonucleic acid; FcεRI, high-affinity IgE receptor I.
